# Computational simulation data using the Lattice Boltzmann method to generate correlations for gas diffusion layer parameters

**DOI:** 10.1016/j.dib.2019.104688

**Published:** 2019-10-19

**Authors:** Mayken Espinoza-Andaluz, Raul Reyna, Yuanxin Qi, Tingshuai Li, Martin Andersson

**Affiliations:** a ESPOL Polytechnic, Escuela Superior Politécnica Del Litoral, ESPOL, Facultad de Ingeniería Mecánica y Ciencias de La Producción, Centro de Energías Renovables y Alternativas, Campus Gustavo Galindo Km. 30.5 Vía Perimetral, P.O. Box 09-01-5863, Guayaquil, Ecuador; b Escuela Superior Politécnica Del Litoral, ESPOL, Facultad de Ingeniería Mecánica y Ciencias de La Producción, Campus Gustavo Galindo Km. 30.5 Vía Perimetral, P.O. Box 09-01-5863, Guayaquil, Ecuador; c Department of Energy Sciences, Faculty of Engineering, Lund University, P.O. Box 118, Lund, Sweden; d School of Materials and Energy, University of Electronic Science and Technology of China, 2006, Xiyuan Ave, West Hi-Tech Zone, Chengdu, Sichuan, China

**Keywords:** Gas diffusion layer, PEFC, Gas-phase tortuosity, Diffusibility, Water-droplet

## Abstract

Analyzing the fluid behavior in complex porous media like gas diffusion layers (GDLs) in polymer electrolyte fuel cells (PEFCs) can be accurately done using the lattice Boltzmann method (LBM). This article shows the data obtained from a study in which diffusion parameters such as porosity, gas phase tortuosity and diffusibility are computed considering simulated porous media [1]. The data were computed when a water drop obstacle is placed inside the GDL domain and the size of the water-drop is varied. Additionally, figures showing the evolution of the flow velocity field are presented alongside graphics that presents the change in local and bulk porosity for each obstacle size. Finally, there is a detailed method explanation concerning the implementation of the lattice Boltzmann method and a general description of computational codes for the domain and obstacle generation as well as the boundary conditions simulation. Data and processes in this article can be exploited in new attempts to solve real case problems in complex mesoscale media.

**Table:** Specifications Table

Subject	Energy
Specific subject area	Energy Engineering and Power Technology, Polymer Electrolyte Fuel Cells
Type of data	Tables, Images, Figures, Codes
How data were acquired	Data was obtained in computer simulations applying the lattice Boltzmann method using an in-house code developed by the authors.
Data format	Simulated & analyzed data, PNG, Code lines
Experimental factors	A velocity field inside a 3D matrix calculated using the lattice Boltzmann method.
Experimental Features	Velocity field graphs, tortuosity, bulk porosity and diffusibility for a small volume inside a complex porous media.
Parameters for data collection	Data was collected under a simulated environment of a gas diffusion layer with a water drop in its domain. The lattice Boltzmann method was employed to obtain the field velocity due to the complexity of the medium as it is too small to be analyzed using computational fluid dynamics.
Description of data collection	Data in tables correspond to bulk parameters of tortuosity, porosity and diffusibility obtained by running the code a number of times while varying the water drop radius inside the medium. These were computed in-code using the velocity field used to generate several image stages.
Data source location	ESPOL (Escuela Superior Politécnica del Litoral) – LabFREE Laboratory of Renewable Sources of Electric Energy. Guayaquil, Ecuador
Data accessibility	Repository name: Computational simulation data using the Lattice Boltzmann Method to generate correlations for gas diffusion layer parameters Data identification number: -Direct URL to data: https://doi.org/10.17632/ynmsvs4pcp.2
Related research article	Author's name: Mayken Espinoza-Andaluz, Raul Reyna, Ayrton Moyón, Tingshuai Li, Martin Andersson. Title: Diffusion parameter correlations for PEFC gas diffusion layers considering the presence of a water-droplet Journal: International Journal of Hydrogen Energy DOI: https://doi.org/10.1016/j.ijhydene.2019.08.144

**Table:** 

**Value of the Data** • The method applied to obtain this data enhances its accuracy in comparison to other computational and experimental methods, making the data shown here more realistic. • The data supplied becomes important to studies that analyze mesoscale models with a complex porous media considering obstacle size randomly placed in its volume. • As there are many different gas diffusion layer structures, these data can be used by studies that aim to compare diffusion parameters between various kinds of porous media or motivate these studies. • Data obtained by this method can be compared to results using different paths. It can spread the interest in analyzing and developing new ways to study mesoscale fluid dynamics. • Several radius of the water drop obstacle in the computed volume that were not displayed in the source article are showed here. The evolution of the fluid velocity field is shown in various successive images for different obstacle sizes.

## 1 Data

**Fig. 1:**
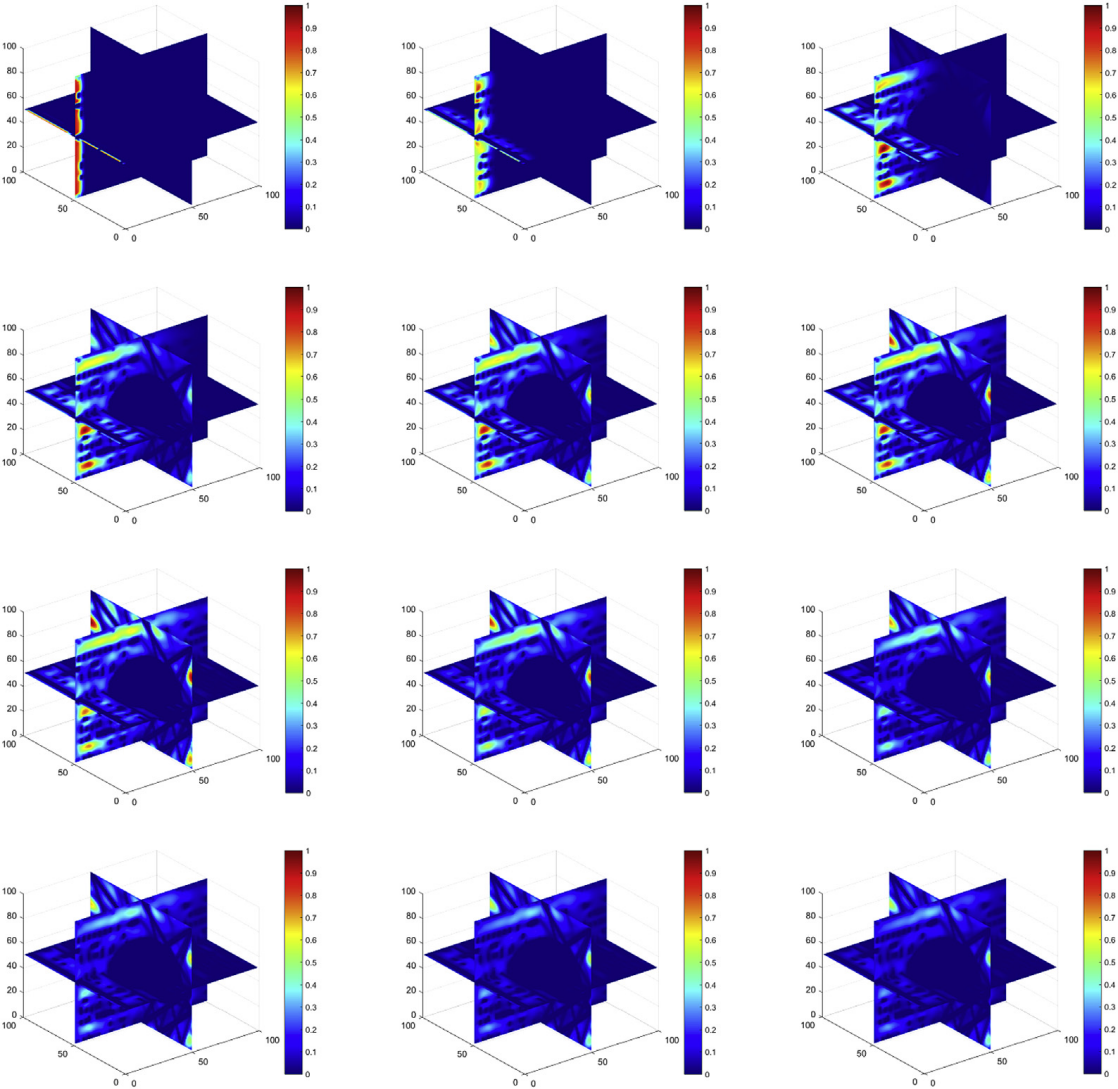
Evolution of the Flow's Velocity Field in porous media as it reaches a steady state condition. Blue represent solid obstacles like the water drop in the middle and fiber materials. Red is for maximum velocity.

The velocity field inside the porous media needs to reach a steady state condition before it can be used to calculate diffusion parameters [1]. Fig. 1 shows how the velocities of the field change after several iterations until they reach the steady state condition, meaning that minimal changes in the medium are occurring. The behavior of the simulated fluid shown in this figure is obtained through the interactions between the moving fluid generated by the Lattice Boltzmann method (LBM) and the packed fibers representing the gas diffusion layer (GDLs). A water drop was placed in the middle, to be easily appreciated. Full display of every step in the flow's velocity field can be found in the dataset attached to the article. Said dataset also has one example that does not cotain a water drop inside.

Porosity, Tortuosity and Diffusibility data were collected after several runs of our code. There were seven iterations for each case to obtain an acceptable standard deviation for each group of data. Bulk Porosity is the average value of local porosities calculated by comparing the void spaces with the whole volume. Tortuosity was obtained by measuring the flow path length and the shortest path length of the medium. Finally, diffusibility is the ratio between the bulk porosity and the tortuosity. Table 1 list the recollection of the parameters computed in the code for all water drop sizes considered.

**Table 1:** Data recollection for gas diffusion layer parameters obtained in several code runs for different water drop radius.

Water Drop Radius [lu]	Diffusion Parameters		
	Bulk Porosity	Tortuosity	Diffusibility
15	0.7850	1.0807	0.7264
	0.7618	1.0875	0.7005
	0.7665	1.0860	0.7058
	0.7857	1.0802	0.7274
	0.7803	1.0792	0.7230
	0.7690	1.0895	0.7058
	0.7746	1.0756	0.7202
20	0.7568	1.0889	0.6950
	0.7493	1.0941	0.6849
	0.7594	1.0766	0.7054
	0.7559	1.0818	0.6987
	0.7600	1.0866	0.6994
	0.7580	1.0821	0.7005
	0.7679	1.1038	0.6957
25	0.7445	1.1111	0.6701
	0.7248	1.1015	0.6580
	0.7197	1.0838	0.6641
	0.7343	1.0791	0.6805
	0.7283	1.0895	0.6685
	0.7211	1.0875	0.6631
	0.7276	1.0968	0.6634
30	0.6873	1.1070	0.6209
	0.6915	1.0891	0.6349
	0.6819	1.0992	0.6204
	0.6992	1.0949	0.6386
	0.6973	1.1059	0.6305
	0.6975	1.1031	0.6323
	0.7042	1.1033	0.6383
35	0.6354	1.1302	0.5622
	0.6464	1.0909	0.5925
	0.6441	1.0902	0.5908
	0.6588	1.1270	0.5846
	0.6386	1.1067	0.5770
	0.6361	1.0967	0.5800
	0.6426	1.1090	0.5794

**Fig. 2:**
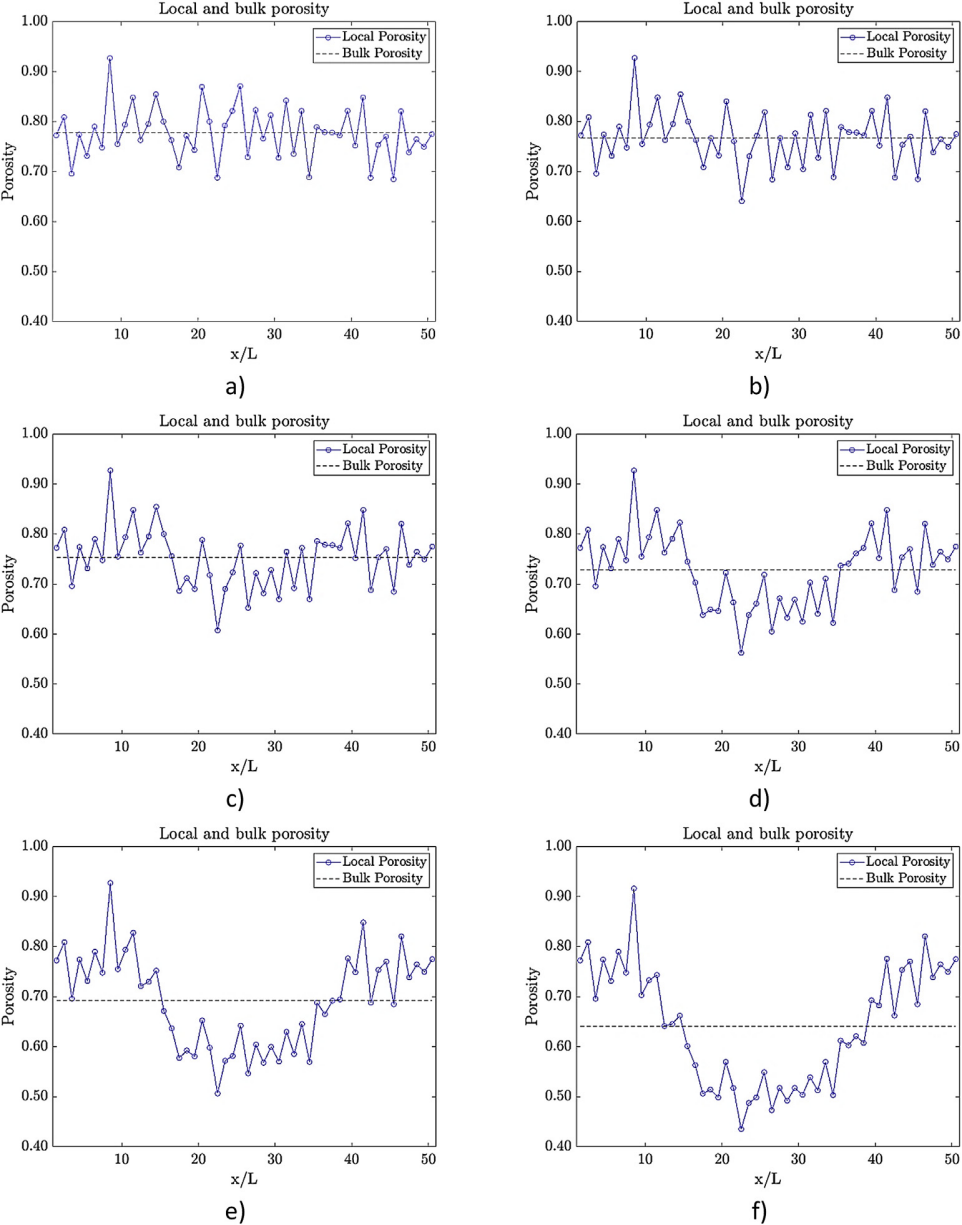
Changes in the local and bulk porosity for a fixed positioned droplet with a varying size. The a) image is for a fiber only domain. From it onwards the radius of the droplet in lu is: b) 15, c) 20, d) 25, e) 30 and f) 35, respectively.

Fig. 2 shows the changes of the local porosity of a medium along the horizontal axis. The images were obtained by setting one constant medium and placing one water drop inside it. The droplet size changes on each iteration showing the variation of the porosity value in every case. The local porosity value was obtained on steps each 2% of the horizontal axis lenght.

## 2 Experimental design, materials, and methods

### 2.1 Lattice Boltzmann method (LBM)

To obtain the computed data of the velocity of the fluid, the LBM was applied. This method works best on complex structures at mesoscale size. First it needs a digitally generated porous medium representing a GDL in which a water drop it is placed. Then it implements boundary condition on the surroundings of the medium and on the interface between fluid and fibers. The computing details are explaining bellow.

### 2.2 Porous media and water drop generation

The first step in the code is the creation of the 3D matrix using zeros to represent voids. In this study a 100 × 100 × 100 zeros matrix was created. This matrix corresponds to a 200 × 200 × 200 μm space inside a GDL [2]. Obstacles in the middle of the matrix correspond to either the GDL's carbon fibers or the water drop. For the fibers, the design was made so the fibers do not deform with the flow, do not have any ends inside the volume and have a cylindrical shape [3]. The water drop was considered as a spherical volume within the matrix. It maintains its shape at all times and never locates outside the GDL. The location of the center of the water drop is randomly placed inside the media while keeping any part of the drop also inside it.

A sample of the code used to generate the media and the water drop is shown in Table 2.

**Table 2:** Sumary of Boundary conditions considered in the data collection.

Description	Code Sample
Matrix generation of the domain	n = 100; m = 100; l = 100; OBSTACLES = zeros(n,m,l);
Randomly placed water droplet	RADIO = [25, 25]; x_positions = randi([26,74],1,2); y_positions = randi([26,74],1,2); z_positions = randi([26,74],1,2);
Loop to generate the water drop in the randomly generated position.	for i = 1:n for j = 1:m for k = 1:l if (sqrt((i-x_positions(index))ˆ2+(j-y_positions(index))ˆ2+(k-z_positions(index))ˆ2)<RADIO(index)) OBSTACLES(i,j,k) = 1; end end end end

### 2.3 Flow field velocity calculation using the Lattice Boltzmann method

To obtain the fluid behavior inside the simulated media, the whole domain has been divided into lattice elements in which the lattice Boltzmann method (LBM) can be applied. All elements interact with the surrounding ones by means of linked velocities. In this 3D case, every element has 19 velocity vectors in different directions.

The method provides a relation between equilibrium and particle distribution for each node. In flow analysis, the equilibrium distribution mainly depends on the local density, velocity and weighting factor for every direction. It also considers collisions in the system and the time it takes to reach a balance. On the other hand, the particle distribution focuses on the position of the node, its velocities and the time lapse to flow in all directions [4,5].

### 2.4 Fluid flow analysis and boundary conditions

The behavior of the fluid depends highly on the equilibrium distribution. In every lattice element, the density and velocity are updated after the collision while the weighting factor of each direction remains constant. This changes the particle velocities in the different directions which interact to the surrounding lattice elements. To maintain the accuracy to real world cases, boundary conditions need to be applied to the interactions between elements [6].

The boundary conditions for the porous media are implemented on the volume limits and in every fluid-solid interface. In this case in which the fluid flows in a single dimension the boundary conditions are divided as presented in Table 3.

**Table 3:** Sumary of Boundary conditions considered in the data obtention.

Applied zone	Boundary Condition	Description
Inlet surface	Von Neumann	The condition is applied as the inlet velocity is known. This is a Velocity Boundary that applies to momentum conservation [7].
Outlet surface	Second Derivative Approximation	As the outlet velocities and pressure are unknown, the conditions are approximated using the velocities of the two previous lattice elements.
Parallel to the flow surfaces	Periodic Boundary	In short, this consideration states that the velocities of the nodes outside the domain are the same as the velocities on the opposite boundary that enters the volume in the same direction.
Solid-Fluid Interface	Bounce Back Condition	When the fluid node collides with a solid interface, the solid reflects the particle in the same action line but opposite direction.

Samples of the boundary conditions codes used are resumed in Table 4.

**Table 4:** Sumary of Boundary conditions considered in the data obtention.

Applied zone	Code Sample
Inlet surface	f2(1:n,1,1:l) = f4(1:n,1,1:l) +rho_in×vo/3;
Outlet surface	f2(1:n,m,1:l) = 2×f2(1:n,m-1,1:l)-f2(1:n,m-2,1:l);
Parallel to the flow surfaces	f3(n,1:m,1:l) = f3(1,1:m,1:l);
Solid-Fluid Interface	for i = 1:n; for j = 1:m; for k = 1:l; if(OBSTACLES(i,j,k) = = 1) temp = f1(i,j,k); f1(i,j,k) = f3(i,j,k); f3(i,j,k) = temp; end; end; end; end;

### 2.5 Solving the algorithm

The algorithm founds the velocity field for each node until the whole system reaches a steady state condition [8]. The first step is the calculation of the distribution function which only happens at the beginning of the code. The phases that form the loop are: a) Collision: It is calculated for the equilibrium distribution at the local node considering the changes in velocity and pressure; b) Streaming: It is the change of the particle distribution functions for the particles around the node; c) Boundary Conditions: These are applied to the nodes of the porous media to maintain the accuracy and stability of the simulation; and d) Steady State Confirmation: Compares the velocities results in every node to the previous velocity until it represents a minimal change. In this simulation the minimal change in velocity is considered as $10^{-3}$.

### 2.6 Diffusion parameters calculation

There are three diffusion parameters shown in the previous data: Bulk Porosity, Gas Phase Tortuosity and Diffusibility [9]. The porosity is calculated by dividing the void spaces volume to the total domain volume. In the simulation, the solid volume is represented by the fibers and the water droplet, so the voids are complement to these. The gas phase tortuosity is obtained after the velocity field is calculated. This is the relation between the flow path length, represented by the velocity sum at each position on every direction, and the shortest path length which corresponds to the velocity sum in the flow direction. Finally, the diffusibility is obtained by the ratio between porosity and tortuosity.
